# Large Language Models in Cardiovascular Prevention: A Narrative Review and Governance Framework

**DOI:** 10.3390/diagnostics16030390

**Published:** 2026-01-26

**Authors:** José Ferreira Santos, Hélder Dores

**Affiliations:** 1 Católica Medical School, Sintra Campus, Estrada Octávio Pato, 2635-631 Rio de Mouro, Portugal; 2 Department of Cardiology, Hospital da Luz Setubal, Luz Saúde, 2900-722 Setubal, Portugal; 3 Department of Cardiology, Hospital da Luz Lisboa, Luz Saúde, 1600-209 Lisbon, Portugal; 4 Associate Laboratory REAL, Comprehensive Health Research Center (CHRC), 1099-085 Lisbon, Portugal; 5 NOVA Medical School, NOVA University Lisbon, 1069-061 Lisbon, Portugal; 6 CoLab Trials, 7000-811 Évora, Portugal

**Keywords:** large language models, artificial intelligence, cardiovascular prevention, risk stratification, clinical decision support

## Abstract

**Background:** Large language models (LLMs) are becoming progressively integrated into clinical practice; however, their role in cardiovascular (CV) prevention remains unclear. This review synthesizes current evidence on LLM applications in preventive cardiology and proposes a governance framework for their safe translation into practice. **Methods:** We conducted a comprehensive narrative review of literature published between January 2015 and November 2025. Evidence was synthesized across three functional domains: (1) patient applications for health literacy and behavior change; (2) clinician applications for decision support and workflow efficiency; and (3) system applications for automated data extraction, registry construction, and quality surveillance. **Results:** Evidence suggests that while LLMs generate empathetic, guideline-concordant patient education, they lack the nuance required for unsupervised, personalized advice. For clinicians, LLMs effectively summarize clinical notes and draft documentation but remain unreliable for deterministic risk calculations and autonomous decision-making. System-facing applications demonstrate potential for automated phenotyping and multimodal risk prediction. However, safe deployment is constrained by hallucinations, temporal obsolescence, automation bias, and data privacy concerns. **Conclusions:** LLMs could help mitigate structural barriers in CV prevention but should presently be deployed only as supervised “reasoning engines” that augment, rather than replace, clinician judgment. To guide the transition from in silico performance to bedside practice, we propose the C.A.R.D.I.O. framework (Clinical validation, Auditability, Risk stratification, Data privacy, Integration, and Ongoing vigilance) as a roadmap for responsible integration.

## 1. Introduction

Cardiovascular disease (CVD) remains the leading cause of mortality worldwide, accounting for approximately 18 million deaths annually and representing a substantial proportion of global disability-adjusted life years [1,2]. Despite significant advancements in identifying modifiable risk factors—such as hypertension, dyslipidemia, diabetes, smoking, obesity and physical inactivity, and the widespread availability of effective interventions, the global burden of CVD continues to escalate.

The 2021 European Society of Cardiology (ESC) guidelines emphasize that most CVD events are preventable through lifestyle modifications and optimal management of risk factors [3]. Nevertheless, a substantial “implementation gap” remains between guideline recommendations and current clinical practice [4]. Recent multinational observational studies demonstrate that only a minority of high- and very-high-risk patients achieve the low-density lipoprotein cholesterol (LDL-C) goals recommended by guidelines, with some cohorts indicating that less than one-quarter of such patients meet targets, despite the availability of potent lipid-lowering therapies [5,6]. Even among patients with established CVD, large-scale registries have highlighted significant gaps in risk factor control, with over 40% experiencing uncontrolled blood pressure and 80% having suboptimal cholesterol levels despite being on medication. Furthermore, more than half remain physically inactive and continue smoking after an event, one-third are obese, while one-quarter have diabetes [7].

Preventive cardiology faces a confluence of critical implementation barriers. Recent evidence highlights that organizational constraints, specifically severe time pressure, workforce shortages, and inadequate clinical decision support, constitute primary bottlenecks in daily practice [8]. These systemic challenges, compounded by the competing priorities of managing multimorbidity, contribute directly to the widespread underuse of objective risk stratification [9]. International surveys indicate that less than half of physicians regularly employ validated cardiovascular (CV) risk calculators, relying instead on clinical judgment which underestimates risk in nearly two-thirds of high-risk patients [10,11]. This gap is largely driven by the perceived complexity of guidelines, with over 60% of physicians reporting that the volume and intricacy of recommendations discourage usage, particularly within time-constrained primary care settings [8].

Even when high risk is identified, therapeutic inertia often persists. Evidence from organizational interventions shows that overcoming hesitation to intensify treatment requires structured, team-based care and regular monitoring systems, rather than relying solely on individual clinician vigilance [6,8]. Crucially, this workload is exacerbated by fragmented data management: essential prognostic information remains dispersed across poorly integrated electronic health record (EHR) sections, forcing clinicians to manually extract critical insights during brief consultations [12,13].

Parallel challenges exist on the patient side, where limited health literacy and inadequate risk perception, often exacerbated by complex medical communication and variable-quality online information, severely undermine adherence [14]. Despite evidence establishing that simplified, plain-language strategies are a ‘clinical necessity’ for effective engagement, such tools remain critically underutilized in routine practice [15].

Large Language Models (LLMs) represent a technological paradigm shift with unique relevance to preventive cardiology. Unlike traditional and more focused artificial intelligence (AI) applications that dominate the field, such as electrocardiogram interpretation or imaging segmentation, LLMs are foundation models trained on vast text corpora [16,17]. This architecture enables them to generate coherent language, synthesize complex information, and perform reasoning tasks without the need for task-specific training [16,17]. These capabilities align remarkably with the core implementation barriers in preventive cardiology, and LLMs offer the potential to automate the extraction of risk factors from unstructured clinical notes, translate technical guideline recommendations into personalized advice, and facilitate the iterative, plain-language communication essential for improving patient health literacy [18,19,20].

However, early evaluations have demonstrated that this potential is accompanied by significant limitations. While LLMs have displayed the capacity to pass medical licensing examinations and provide generally appropriate prevention advice, critical safety concerns persist [21]. Studies evaluating generative artificial intelligence (AI) responses to CV prevention scenarios found that, while broadly accurate, the outputs often lacked the clinical nuance required for individualized treatment intensity decisions [22]. Furthermore, comparative analyses have revealed variability in accuracy across different models and languages, alongside the persistent risk of “hallucinations”, the generation of plausible but factually incorrect information [22,23]. These reliability issues constitute a major barrier to clinical deployment that must be rigorously addressed.

Although several recent reviews have examined the applications of LLMs in medicine broadly or cardiology in general, few have addressed the specific challenges of CV prevention [22,24,25]. Current literature predominantly focuses on diagnostics or imaging analysis, frequently overlooking the longitudinal and behavioral complexities inherent to risk factor management [26]. Furthermore, the transition from theoretical capability to clinical utility is hindered by a paucity of robust implementation frameworks, specifically those capable of reconciling generative AI with established risk scores and rigorous safety governance [27,28].

This review examines the practical use of LLMs in CV prevention to ensure they help reduce the global CVD burden instead of adding unnecessary complexity. We synthesize current evidence across three functional domains: (1) patient applications aimed at enhancing health literacy and supporting behavior change; (2) clinician applications designed to expand risk stratification, early diagnosis of CVD, guideline adherence, and workflow efficiency; and (3) system applications that operate at the infrastructure level to enable automated data extraction, registry construction, and quality surveillance. Building on these insights and a critical analysis of safety risks, we propose a conceptual framework (C.A.R.D.I.O.) for the responsible integration of LLM-based tools into preventive cardiology workflows.

## 2. Methods and Literature Search

This work was conducted as a comprehensive narrative review, with data extraction and synthesis organized around the three core application domains (patient, clinician, and system) outlined in the introduction.

We searched electronic databases (PubMed/MEDLINE, Embase, Scopus) and preprint repositories (arXiv, medRxiv) for English-language articles published between 1 January 2015 and 30 November 2025. The search strategy combined terms related to LLMs and generative AI (e.g., “large language model*”, “LLM*”, “generative AI”, “ChatGPT”, “transformer*”) with cardiovascular and prevention-related terms (e.g., “cardiovascular”, “cardiology”, “primary prevention”, “secondary prevention”, “risk factor*”, “risk stratification”, “clinical decision support”).

The exact syntax was adapted for each database. Consistent with narrative review methodology, evidence selection relied on author expertise to identify relevant literature rather than systematic screening protocols. Additional records were identified through manual cross-referencing and backward citation searching. We included original studies describing the development, validation, or implementation of LLM-based or closely related generative language tools in cardiology or cardiovascular prevention, as well as narrative reviews, position papers, and consensus documents from major professional societies addressing AI or LLMs with explicit implications for CV prevention. Foundational computer science papers on transformer architectures and general-purpose LLMs were included when directly relevant to clinical or health-system applications. We excluded studies focusing exclusively on computer vision applications without a substantive text-based or language-model component, and purely technical descriptions of non-medical AI models without clear relevance to healthcare or prevention.

**Figure 1:**
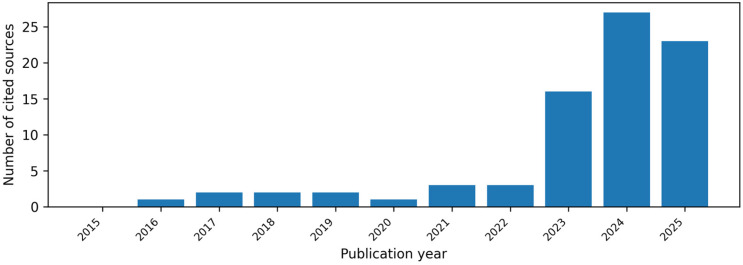
Temporal distribution of cited sources within the review period.

Records were reviewed at the title/abstract level for relevance to the review objectives, and full texts were consulted where appropriate. Selection was guided by the three predefined application domains and the objective of identifying influential and representative evidence in this rapidly evolving field. Initial screening was conducted by J.F.S.; uncertainties were discussed with H.D. and resolved by consensus. Given the rapid pace of AI development, particular emphasis was placed on work published after the introduction of the transformer architecture in 2017, although earlier contributions were retained when necessary to contextualize current LLM-based approaches [29]. To contextualize this rapidly evolving evidence base, we summarize the temporal distribution of cited sources within the review period in Figure 1; the concentration of publications after 2023 reflects the post–ChatGPT acceleration of research activity. Because of the heterogeneity of study designs (technical benchmarks, observational studies, pilot interventions, conceptual and policy papers), no formal risk-of-bias assessment or meta-analysis was undertaken. Instead, findings were synthesized narratively and organized according to the three levels of applications. These findings informed the development of the conceptual governance framework (C.A.R.D.I.O.) proposed in the final section.

## 3. Fundamentals of Large Language Models for Clinicians

**From Calculators to Probabilistic Engines.** The emergence of LLMs represents a transformative advancement in AI, dramatically demonstrated with ChatGPT’s release in late 2022 revealing unprecedented conversational capabilities [30]. To understand the utility of these tools in preventive cardiology, clinicians must clearly distinguish them from the tools currently employed in practice. Traditional risk tools, such as the SCORE2 or Pooled Cohort Equations, are deterministic: they apply fixed regression equations to a set of variables to produce a consistent numerical output [3,31]. Similarly, “narrow” AI applications are constrained to specific tasks like electrocardiogram interpretation [26,32]. In contrast, LLMs are probabilistic engines trained on petabytes of text [17,33]. Rather than “calculating” risk, they generate language by predicting the next statistically likely token (word fragment) based on context [16]. Through the scaling of both model parameters and training data, these systems have developed “emergent” capabilities, that include sophisticated question answering, document summarization, and multi-step reasoning—abilities that were not explicitly programmed but arose from patterns learned across billions of text examples [34,35]. This architecture allows them to process unstructured clinical narratives that break traditional software, but it also introduces the risk of generating plausible yet factually incorrect statements, known as “hallucinations” [17,21,36,37]. To aid clinicians in navigating this new landscape, key technical concepts and their practical implications are defined in Table 1.

**Development and Training Stages.** The creation of a clinically applicable LLM typically follows three progressive stages. First, during pre-training, the model is exposed to vast text corpora from the internet. It acquires a broad comprehension of medical language and concepts but lacks clinical specificity or safety constraints [16,33,36]. Second, in supervised fine-tuning, the model is adapted to the medical domain [38]. Developers train it on curated datasets of clinical guidelines, question–answer pairs, and de-identified notes, effectively transforming broad linguistic knowledge into focused medical expertise [16,33,36,38]. The final stage, alignment, often uses reinforcement learning from human feedback [36]. Human evaluators rate model outputs for safety, training the model to refuse harmful queries. However, alignment remains imperfect; models can still be manipulated (“jailbroken”) and may generate incorrect medical information with high confidence [36,39].

**Deployment Strategies.** In practice, clinicians will encounter three primary deployment strategies. General-purpose models (e.g., GPT-4) offer flexible reasoning but may generate medically incorrect advice that diverges from guidelines [17,21]. Domain-adapted models (e.g., Med-PaLM 2) are fine-tuned on biomedical literature for higher accuracy but often remain “frozen” in time, unable to incorporate new evidence [38,40]. Composite systems represent the most reliable approach for prevention; they embed LLMs into broader workflows—using retrieval-augmented generation (RAG) to cite external guidelines or integrating with electronic health records (EHR)—to improve clinical utility and reduce errors [39,41,42].

**Capabilities in Cardiovascular Prevention.** Within this framework, LLMs offer four capabilities relevant to preventive cardiology:

- Information Synthesis: Consolidating fragmented patient histories into concise summaries [18,38].
- Risk Factor Extraction: Identifying unstructured variables (e.g., family history, symptoms) from free-text notes [25,28,42].
- Guideline Translation: Converting complex technical recommendations into personalized care pathways [21,37].
- Generative Communication: Creating plain-language explanations to support patient health literacy [14,43].

**Evaluation and Risks.** As LLMs enter clinical use, evaluation must move beyond standardized exams to focus on guideline concordance, calibration, and robustness [21,34,38]. True readiness requires prospective assessment in real-world workflows to monitor for automation bias, where clinicians uncritically accept AI outputs [20,27,36]. Despite their potential, significant risks remain. Models may hallucinate, fabricating trials or misquoting guidelines [21,23,36,37]. Limited context windows may cause models to “forget” relevant history in long records [35,39]. Finally, without retrieval mechanisms, models may provide outdated recommendations or amplify biases present in training data, potentially affecting care for under-represented groups [33,37,44].

## 4. Current Evidence on Large Language Models for Cardiovascular Prevention

**Figure 2:**
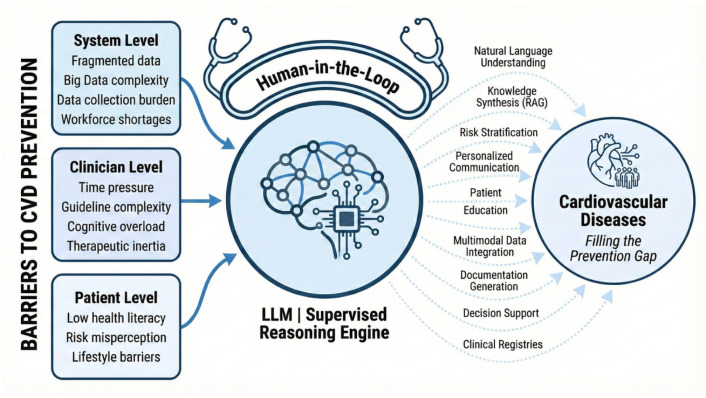
Large language model–augmented approach to address barriers and fill the prevention gap in cardiovascular disease.

Having defined the technical architecture and capabilities of LLMs, we now examine the emerging evidence supporting their translation into preventive cardiology. The transition from general-purpose chatbots to medical-grade tools requires rigorous evaluation against the specific challenges of risk factor management [17,20]. To structure the heterogeneous evidence base, we organize the current landscape of literature around the three key stakeholders in the prevention pathway: the patient, the clinician, and the health system (Figure 2).

While the theoretical potential of these models is vast, current practice is defined by relatively narrow applications that target specific barriers within CV prevention workflows [20,22]. To structure this heterogeneous evidence base, we categorize existing applications into three functional domains: (1) patient applications aimed at enhancing health literacy and supporting behavior change; (2) clinician applications designed to expand risk stratification, guideline adherence, and workflow efficiency; and (3) system applications that operate at the infrastructure level, including automated data extraction, registry construction, quality surveillance, and multimodal prognostic modeling. Together, these domains illustrate how LLM-based tools begin to address the “implementation gap” in CV prevention by informing patients, supporting clinicians, and enabling scalable, system-level optimization of preventive care. A summary of key capabilities, benefits, and risks across these three domains is provided in Table 2.

### 4.1. Patient Applications

The most immediate application of LLMs involves their direct use by patients for medical information retrieval and lifestyle support. Unlike traditional search engines or rigid rule-based chatbots, LLMs function as interactive agents capable of synthesizing information rather than merely retrieving static links [18,20]. This capability has prompted extensive investigation into their utility for improving health literacy and facilitating shared decision-making regarding modifiable risk factors [17,23].

**Information accuracy and safety.** The primary determinant of clinical utility in this domain is the factual accuracy and safety of the generated advice. A recent systematic review synthesized evidence from 35 observational studies evaluating LLM applications in CVD, the majority of which focused on patient education and risk factor management [22]. The review found that current models, particularly versions of ChatGPT (OpenAI), demonstrate a high capacity for answering common patient inquiries. Across multiple studies, the aggregated evidence suggests that LLMs provide “accurate, comprehensive, and generally safe” responses to questions covering diverse domains, including heart failure, atrial fibrillation, traditional risk factors such as hypertension and diabetes, and lifestyle interventions [22].

This broad reliability is exemplified by early pivotal studies evaluating LLM responses to fundamental prevention questions. Cardiologists graded the model’s outputs as “appropriate” in approximately 84% of cases, noting that advice on diet, exercise, and lipid management was largely consistent with current guidelines [45]. However, this high baseline accuracy is accompanied by significant limitations. Models often suffer from “temporal obsolescence”, lacking awareness of recently published guidelines, novel drug approvals, or new procedures [22,37,38]. Furthermore, performance variability has been noted across languages, with some studies showing accuracy drops when queries are posed in non-English languages [22]. While dangerous advice is rare, the persistent risk of hallucination and the confident fabrication of references remain a safety barrier for unsupervised use [21,22,36].

Crucially, there is a distinction between “broad accuracy” and “clinical precision”. While models are generally correct on high-level concepts, they frequently lack the nuance required for personalized treatment decisions. For instance, an LLM may correctly identify the general benefit of statins but fail to distinguish between the varying intensity targets for primary versus secondary prevention, or neglect to tailor advice for patients with specific comorbidities [22,45].

**Communication quality and health literacy.** Beyond factual content, the way in which information is conveyed is critical for patient engagement. In this regard, LLMs have demonstrated a notable capacity to outperform physicians in communication tone. In direct comparisons of responses to patient inquiries, evaluators have rated LLM-generated answers as significantly more empathetic and higher in quality than those drafted by clinicians [46]. By utilizing validating language and avoiding the brevity that often characterizes time-pressured medical correspondence, these models offer a potential solution to the “empathy deficit” frequently cited in digital health interactions [46].

Nevertheless, this empathetic answering is often undermined by a paradox in technical accessibility. While the tone is supportive, the default linguistic complexity often exceeds the health literacy of the average patient [22]. CV prevention relies on the comprehension of nuanced concepts, yet evaluations of LLM-generated education materials consistently find them written at a high school or college reading level (11th–13th grade), far surpassing the recommended 6th-grade standard [47]. Although LLMs possess the capability to simplify text when explicitly prompted, this requires user initiative. Without specific prompt engineering (e.g., “explain this to a 12-year-old”), the inherent complexity of LLM outputs remains a significant barrier to equitable access, particularly for vulnerable populations [48].

**Lifestyle behavior and risk factor modification.** The frontier of patient-facing LLM applications lies in their potential to promote active behavior change. Theoretical frameworks suggest that the generative nature of these models enables the simulation of motivational interviewing, delivering unique, context-aware responses that address specific patient barriers [49,50]. Unlike static apps restricted to pre-scripted reminders, an LLM-based coach can theoretically engage in iterative dialogue, exploring the reasons for medication non-adherence or proposing personalized dietary and exercise adjustments. While pilot studies targeting general lifestyle risk factors have shown promise, robust evidence supporting the translation of these capabilities into effective CV interventions remains limited [49,50].

Taken together, current evidence indicates that patient-facing LLMs can provide generally accurate, guideline-concordant, and empathetic information across common CV topics [22,45,46]. However, their safe use remains constrained by static training data, variable performance across languages and literacy levels, the potential for hallucinated content, and the absence of prospective trials demonstrating improvements in risk factor control or clinical events [21,22,36,37,38,49,50,51]. At present, these systems are best regarded as adjuncts for patient education and engagement rather than validated standalone advisors for individualized CV prevention [22,45].

**Risks, limitations, and evidence gaps in patient applications.** Ultimately, while patient education represents the most extensively studied application, accounting for nearly three-quarters of the current literature, there is a paucity of prospective trials evaluating its impact on clinical endpoints [51]. The existing evidence base remains predominantly evaluative, focusing on the quality of textual outputs rather than interventional efficacy. Consequently, the specific capability of patient-facing LLMs to drive sustained improvements in risk factor control and prevent CV events remains to be established through rigorous clinical trials [22].

### 4.2. Clinicians Applications

The highest-yield applications of LLMs in preventive cardiology arguably lie in improving clinical workflows and clinician performance. In this domain, LLMs function not merely as encyclopedic references but as “reasoning engines” capable of processing unstructured data to support diagnostic and therapeutic decision-making [17,18]. Unlike “narrow” AI tools designed for specific tasks, clinician-facing LLMs can operate as broad-spectrum support systems.

**Guideline retrieval and reference consultation.** The sheer volume of CV prevention guidelines, spanning hypertension, dyslipidemia, diabetes, and lifestyle modification, alongside secondary prevention protocols, presents a cognitive challenge for time-pressured clinicians [3,31]. A primary utility of LLMs is the instant retrieval and synthesis of these complex recommendations. Initial evaluations of LLM performance on standardized medical examinations and cardiology-specific board questions have been promising, with general-purpose models often achieving passing scores without specialized training [16,52].

Recent studies evaluating LLMs against ESC guidelines demonstrate that these models can accurately interpret Class I and III recommendations in over 80% of selected clinical scenarios [21]. Similarly, when presented with vignette-based queries regarding CVD prevention, widely available LLMs have demonstrated the capacity to provide advice that is “broadly appropriate” and concordant with guidelines in most cases [53,54]. Recognizing this potential, professional societies are increasingly exploring RAG systems, tools that ground LLM answers specifically in verified guideline texts, to allow clinicians to query trusted repositories directly. This approach mitigates the risk of hallucination inherent in open-ended models, a strategy exemplified by the ESC Chat recently made available to medical professionals [55].

**Decision support and risk stratification.** A critical distinction must be made between guidelines and literature recall and clinical decision making. In preventive cardiology, the ability to recite a guideline is less valuable than the ability to apply it to a specific patient. LLMs often struggle with the nuance required for complex decision-making [56,57,58]. Evidence suggests that when presented with multimorbid scenarios, LLMs may default to aggressive, standard-of-care recommendations that fail to account for competing risks, frailty, or pill burden [57,59,60]. Furthermore, model outputs often lack the specificity required for immediate action; for instance, a model might suggest “initiating high-intensity statin therapy” without specifying the agent or dosage appropriate for a patient’s specific renal function or drug–drug interaction profile [61].

A common misconception is that LLMs, being computational tools, are inherently capable of mathematical risk prediction. In reality, the token-prediction architecture of LLMs makes them unreliable arithmeticians, and studies benchmarking LLMs on medical calculations reveal high error rates [41]. Consequently, current stand-alone LLMs should not be used to replace deterministic risk calculators. Instead, the emerging approach involves a hybrid architecture known as “function calling.” In this workflow, the LLM extracts the necessary variables (e.g., age, systolic blood pressure, smoking status, HDL levels) from unstructured clinical notes and transmits this data to an external interface to perform the calculation [24,42]. The LLM then receives the precise result and generates a plain-language interpretation for the clinician. While this architectural approach is currently being validated in broader medicine contexts, it represents a logical evolution for CV prevention, effectively bridging the gap between unstructured narrative data and rigid risk scoring systems [24,37,42].

**Documentation, summarization, and administrative workflow.** Perceived time pressure is a leading barrier to the implementation of preventive strategies [9,12]. Clinicians frequently cite the difficulty of synthesizing a patient’s CV history from the EHR as a deterrent to performing comprehensive risk assessments. LLMs integrated into the EHR infrastructure offer a solution to this “information overload.” Models trained on clinical notes have demonstrated the ability to summarize complex, longitudinal patient histories into concise “problem lists” with high accuracy in pilot studies [42].

In the context of prevention, such tools can automatically scan years of progress notes to flag relevant history that might be missed during a brief consultation. Furthermore, LLMs can act as a safety net for “diagnostic rescue” by identifying overlooked data that are not formally coded in the problem list (e.g., scanning historical imaging reports to identify incidental coronary calcification) [25,41]. By presenting this summary information directly to the clinician at the point of care and contextualizing a forgotten report against the patient’s current profile, these tools may reduce the cognitive load required to identify high-risk patients who are frequently under-identified and undertreated [8,25].

One of the most transformative clinician-facing applications is “ambient listening” technology, which is seeing rapid adoption in hospital settings [62,63]. These systems utilize automatic speech recognition paired with LLMs to transcribe clinician-patient encounters in real time and generate structured clinical documentation [63]. For preventive cardiology, the implications of ambient AI extend beyond mere convenience. By automating the creation of clinical notes, these tools theoretically liberate the clinician to maintain eye contact and engage in the nuanced dialogue required for motivational interviewing and shared decision-making. Early trials suggest that ambient AI tools can significantly reduce time spent on documentation and are associated with reduced measures of burnout [64,65,66]. Furthermore, these systems can be configured to automatically generate patient-friendly “After Visit Summaries” that translate the consultation’s medical jargon into clear, actionable steps for lifestyle change, thereby reinforcing the prevention plan discussed during the visit [12,65,67].

**Risks, limitations, and evidence gaps in clinician applications.** Despite these potential benefits, the deployment of clinical LLM applications introduces novel safety risks that require rigorous governance. The most prominent risk remains the generation of confident but false information (“hallucinations”) [21,36]. In the context of guidelines, an LLM might invent a recommendation or fabricate a citation to support a treatment decision. Additionally, as already previously noted, standard LLMs suffer from “temporal obsolescence” and are frozen at the time of their training [37]. A model trained prior to recent updates will be unaware of new guidelines or novel therapies, necessitating that clinicians maintain independent, up-to-date knowledge to verify model outputs.

Finally, as models become more reliable, there is a paradoxical risk of “automation bias,” where clinicians may become complacent, accepting AI-generated risk assessments or treatment plans without scrutiny [20,24]. This is particularly dangerous in preventive cardiology, where subtle patient preferences or social determinants of health, which the model may not appreciate, often dictate the success of a long-term treatment plan [23]. Furthermore, the use of ambient listening raises undefined legal questions regarding liability if an AI-generated note omits a critical symptom that leads to a missed diagnosis [68].

In summary, LLM applications in direct medical care hold the potential to act as powerful extensions of the clinician, handling the computational burden of data extraction, guideline retrieval, and documentation [20,41]. This technological support may allow the physician to focus on the uniquely human task of personalized risk negotiation [15,18]. However, these applications lack the reliability to function as autonomous decision-makers, necessitating a “human-in-the-loop” workflow where every AI-generated suggestion is rigorously verified by a supervising clinician [34,39].

### 4.3. System Applications

System-facing applications of LLMs operate at the level of health systems, payers, and research infrastructures rather than individual encounters. Outputs from these tools are typically not presented directly to patients or clinicians but are used to populate registries, drive performance dashboards, support population health programs, and underpin real-world research. Within cardiovascular prevention, such “back-end” uses are central to addressing structural contributors to the implementation gap by enabling scalable phenotyping, continuous quality assurance, and integration of heterogeneous diagnostic and prognostic data [4,27,42].

**Automated population phenotyping and data extraction.** Effective system-level prevention depends on the accurate identification of high-risk cohorts across large populations. Traditional methods rely heavily on structured fields such as diagnosis codes and problem lists, which frequently fail to capture nuanced risk modifiers like family history, statin intolerance, functional limitations and psychosocial factors, that are often recorded only in free text [25,42]. System-facing transformer models leverage dense contextual representations to process large volumes of clinical narratives and can detect complex disease states and risk phenotypes with sensitivity that exceeds earlier rule-based extraction approaches [33,42].

Foundational work with transformer encoders pretrained on EHR data, such as Med-BERT, has demonstrated superior performance in phenotyping tasks compared with conventional models, learning clinically meaningful patterns from sequences of diagnostic and procedural codes [69]. When fine-tuned on cardiology narratives, such architectures achieve high fidelity in extracting core CV risk factors directly from unstructured notes, effectively creating “computable phenotypes” independent of billing codes [25,42]. Applications in heart failure, where models have been used to characterize New York Heart Association (NYHA) class and identify precipitants of decompensation from discharge summaries, provide proof of concept for this paradigm [70,71,72]. Translated to prevention, these approaches offer a means to capture uncodified prognostic variables, including frailty descriptors, barriers to medication adherence, and social determinants of health, at scale [73]. A key organizational opportunity is the repurposing of these pipelines from episodic analyses to automated, near-real-time registries. By continuously scanning clinical narratives for predefined risk patterns, health systems could systematically detect care gaps invisible to structured queries alone and maintain up-to-date lists of high-risk patients requiring intensified prevention [8,27].

**Quality assurance and clinical registry automation.** Automated phenotyping naturally feeds into quality assurance and registry maintenance. Historically, quality measurement in cardiovascular prevention has relied on manual chart review or analyses of structured administrative data, approaches that are labor-intensive, delayed, and prone to misclassification [21,70,74]. Early work with rule-based natural language processing (NLP) showed that key quality metrics, such as documentation of discharge instructions or left ventricular ejection fraction (LVEF), could be extracted from narrative text with performance comparable to human abstractors [75,76].

Modern NLP and LLM-based architectures extend this concept by refining diagnostic coding, correcting misclassified cases, and linking narrative descriptors to prescription and laboratory data [70,77]. This enables automated detection of complex “care gaps,” such as patients with established atherosclerotic cardiovascular disease who lack statin therapy or individuals with persistently elevated blood pressure despite frequent clinical encounters [74]. In principle, these systems allow a transition from periodic sample-based audits to continuous, system-wide surveillance of guideline adherence and attainment of prevention targets. High-resolution registries derived in this way would be valuable both for internal quality improvement and for benchmarking across institutions [78].

**Resource optimization and cost efficiency.** Health systems face a growing mismatch between the number of individuals requiring long-term CV risk management and the available specialist workforce [1,4]. In this context, system-level AI can function as an intelligent triage and planning engine [27,62]. By analyzing longitudinal records, models can stratify populations not only by estimated risk but also by complexity profiles, distinguishing those needing referral to specialized preventive cardiology services from those who can be managed in primary care [25,77].

Beyond triage, LLM-based pipelines can reduce the marginal cost of high-resolution population surveillance by automating labor-intensive tasks such as chart abstraction for registries, manual coding of discharge summaries, and repeated screening of eligibility criteria for disease management programs [70,75,76]. This automation could allow health systems to monitor large cohorts continuously, identify care gaps earlier, and reallocate human effort from data handling to clinical decision-making and patient engagement [27,62].

**Big data, multimodal integration, and precision medicine.** Conventional CV prevention strategies are inherently minimalist, relying on a limited set of variables to estimate risk. While such scores perform well at the population level, they omit most of the biological and environmental information that shapes individual trajectories [27,77]. The next frontier for system-facing AI is “deep phenotyping”, integrating diverse data streams into a high-dimensional representation of CV risk [25,27].

Achieving this requires expansion of the input layer beyond structured EHR data to encompass: (1) multi-omics information (e.g., genomics including polygenic risk scores, proteomics, microbiomics); (2) environmental and social exposures, quantified via geospatial data on air pollution, noise, and neighborhood deprivation; and (3) continuous physiological data from wearables, capturing heart rate variability, sleep patterns, and physical activity [27,77]. Additionally, narrow AI models play a crucial intermediate role by extracting high-value features from complex modalities, such as radiomic markers from imaging (e.g., perivascular fat attenuation on computed tomography angiography) or signal-based biomarkers from electrocardiograms (e.g., AI-estimated biological age or inferred atrial fibrillation risk) [26,32,74]. Within a system-level architecture, LLMs can serve as a semantic orchestration layer that links these heterogeneous outputs with clinical narratives, producing a unified, interpretable risk profile [20,42]. This multimodal integration underpins visions of precision prevention and “digital twin” models in cardiology, although significant challenges remain in terms of data interoperability, privacy, and regulatory oversight [27,39,51].

**Novel prognostic systems.** Transformer-based and LLM-related architectures also enable a transition from static, one-off risk scores to dynamic prognostic systems that model full patient trajectories. Rather than summarizing history into a single baseline snapshot, these models encode longitudinal sequences of diagnoses, prescriptions, laboratory trends, and textual notes into high-dimensional embeddings. These representations can then be used to predict future cardiovascular events with greater temporal resolution and discriminatory performance than traditional regression approaches [25,27,67].

Work with Med-BERT has shown that transformers pretrained on structured EHR sequences outperform conventional deep-learning models on various disease prediction tasks by capturing contextual relationships between clinical events [69]. Building on this, the TRisk model has applied transformer-based learning to longitudinal datasets in primary prevention, achieving a C-index of 0.910 and substantially outperforming standard guideline-recommended risk scores for cardiovascular risk estimation [79]. Similarly, multimodal frameworks such as “PreCog” have begun to combine clinical transformers with high-fidelity diagnostic data, including cardiac magnetic resonance imaging features, polygenic risk, and metabolic profiles, identifying subgroups with markedly elevated relative risk of incident coronary artery disease compared with baseline estimates [77].

In parallel, investigators have evaluated whether general-purpose LLMs can perform risk stratification using structured inputs. When tested against conventional American College of Cardiology/American Heart Association (ACC/AHA) risk scores using cohorts such as the UK Biobank and KoGES (Korean Genome and Epidemiology Study), GPT-4 has demonstrated broadly comparable discrimination, suggesting that general LLMs can interpret risk variables and approximate standard calculators when appropriately prompted [80]. Nevertheless, even when discrimination is improved, calibration, clinical utility, and safety remain critical concerns, particularly when models are applied across populations and healthcare settings different from those used for development [37,39,57,59].

**Bridge to research and clinical trials.** System-level LLM applications also intersect with CV research, especially in real-world evidence generation and optimization of clinical trial workflows. Trial recruitment is a persistent bottleneck that delays evaluation and dissemination of new preventive therapies. By parsing complex eligibility criteria and matching them against unstructured EHR data, LLM-based systems can identify potentially eligible participants at scale and across multiple sites, streamlining both feasibility assessments and active recruitment [78].

More broadly, LLM-enriched registries and phenotyping pipelines can support observational studies of preventive therapies in populations that more closely resemble routine practice than highly selected trial cohorts [27,42]. Such systems may help characterize patterns of uptake of novel interventions, quantify therapeutic inertia in high-risk primary prevention, and evaluate heterogeneous treatment effects across subgroups and health systems [25,27]. However, these applications are sensitive to the accuracy of labels, stability of underlying data distributions, and potential biases introduced by algorithmic processing [39,44,57]. Regulatory authorities are increasingly attentive to the provenance, transparency, and auditability of AI-derived real-world evidence, implying that LLM-based research workflows will require rigorous validation, version control, and explicit documentation before their outputs can inform guidelines or regulatory decisions [51,74].

**Risks, limitations, and evidence gaps in system applications.** Across system-facing use cases, several shared limitations constrain translation into routine cardiovascular prevention. Most reported implementations remain retrospective, single-center, or proof-of-concept analyzes, with limited evidence on external validity across institutions, healthcare systems, and demographic subgroups [22,51,57]. Model performance is tightly coupled to documentation practices and data quality; settings with sparse or low-quality clinical notes may see degraded performance, raising concerns that automated phenotyping and quality metrics could systematically under-represent disadvantaged or under-resourced populations [39,44]. The opacity of LLM-derived representations complicates governance, as it may be difficult to distinguish genuine differences in care quality from algorithmic artefacts when interpreting performance dashboards or risk stratification outputs [20,74]. For advanced multimodal and prognostic systems, issues of calibration, drift over time, and robustness to shifts in practice patterns remain incompletely characterized, and prospective impact evaluations are missing [39,51,59]. Finally, large-scale linkage and processing of genomic, environmental, and behavioral data raise substantial concerns regarding privacy, consent, and acceptable secondary uses of data [17,68]. Collectively, these factors indicate that while system-facing LLM applications are central to the long-term vision of precision, population-level cardiovascular prevention, their safe deployment will require staged implementation, rigorous validation, and explicit fairness and governance frameworks [51,62,74].

## 5. Future Directions: From Hype to Clinical Utility

Despite rapid advances in model capability, current evaluations of LLMs in CV prevention remain dominated by retrospective benchmarks, vignette studies, and technical demonstrations rather than interventional trials embedded in care pathways [22,51,57]. The next phase of research must therefore shift from describing what models can do in principle to demonstrating what carefully governed systems achieve for patients, clinicians, and health systems in practice [26,62,74].

To bridge this gap, the field must undergo a fundamental translational shift from in silico performance to bedside utility [22,51]. The research agenda must move beyond evaluating text quality toward measuring clinical impact through prospective, pragmatic trials [51,62]. While the capacity to summarize patient history or explain guidelines is valuable, true utility must be demonstrated by tangible improvements in real-world care [27,42]. Future investigations should prioritize a spectrum of endpoints, ranging from intermediate biomarkers to hard clinical outcomes, including reductions in myocardial infarction and CV mortality [25,77]. Additionally, researchers must evaluate process metrics that reflect the quality of preventive care, such as medication adherence, the utilization rates of validated risk scores, and the appropriateness of specialist referrals among others [9,27].

Equally critical is the rigorous evaluation of safety and trials should explicitly monitor for “automation bias”, the tendency for clinicians to uncritically accept AI-generated recommendations, particularly in complex, multimorbid cases [20,24,58]. Furthermore, for patient-facing applications, surveillance must extend to unintended behavioral harms, quantifying risks such as inappropriate reassurance, delayed help-seeking, and the propagation of subtle but clinically significant misinformation [21,23,34].

In parallel, technological development must evolve from passive information retrieval chatbots to more sophisticated “agentic workflows” and composite systems [81]. Building on the function-calling capabilities, in this emerging paradigm, the LLM functions not merely as a text generator but as an orchestrator authorized to perform a series of constrained, auditable actions via function calling [24,81]. Rather than simply suggesting that a risk assessment be performed, an agentic system could autonomously extract relevant variables from unstructured notes, execute a validated SCORE2 calculation through an API, query a drug–drug interaction database, and prepare a draft referral to a lipid clinic, all for clinician review and signature [41,81]. The clinician’s role shifts from manual data entry to executive verification of extracted data, calculations, and proposed orders [62,64]. While these capabilities are technically available, healthcare applications integrating function calling with validated clinical calculators remain at an early research stage [81].

To ensure safety and consistency, such systems must rely on RAG, thereby anchoring outputs in versioned, authoritative repositories, such as current ESC or AHA guidelines and institutional protocols, rather than the model’s opaque internal training data or uncurated web sources [37,55]. Future evaluation frameworks will need to consider the behavior of the entire composite workflow, including robustness to atypical inputs and strict adherence to predefined safety boundaries [51,57].

Finally, the clinical translation of these tools must rigorously address equity, regulation, and global scalability [17,44]. There is a substantial risk that models trained on unrepresentative data will not only reproduce but amplify historical disparities in CV care [44,82]. Future validation studies must therefore necessitate the reporting of performance stratified by sex, ethnicity, and, where possible, socioeconomic status and language, scrutinizing calibration and error patterns in under-represented groups [39,82].

From a governance standpoint, regulatory frameworks must treat LLM-based tools for CV prevention as adaptive clinical decision support systems rather than static software [51,74]. This requires adopting a total product lifecycle perspective that includes pre-deployment validation, transparent documentation of intended use, structured processes for updating knowledge bases as guidelines evolve, and continuous post-deployment monitoring for model drift or unsafe performance [57,83]. If these conditions are met and systems are rigorously validated across diverse populations, LLMs could act as transformative force-multipliers in resource-limited environments [84]. By equipping primary care clinicians with accessible, guideline-based decision support and automated synthesis of complex preventive recommendations, they may help democratize access to specialist-level expertise and partially mitigate workforce shortages that currently hinder effective cardiovascular risk management outside tertiary centers [4,8,84].

## 6. A Conceptual Framework for Safe Clinical Translation

**Figure 3:**
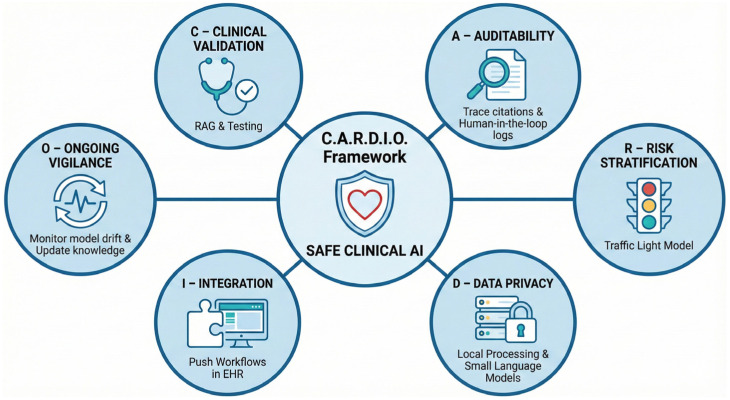
C.A.R.D.I.O. governance framework for safe clinical translation of large language models in cardiovascular prevention.

To move LLMs from experimental tools to reliable clinical instruments, ad hoc adoption must be replaced with structured governance. The unique risks posed by generative AI, specifically hallucination, non-determinism, and temporal obsolescence, require a departure from traditional software regulation. Building on the evidence synthesized in this review, we propose the **C.A.R.D.I.O. Framework**: a pragmatic roadmap designed to align generative AI with the rigor of preventive cardiology (Figure 3). The framework was developed through author synthesis of evidence gaps and safety concerns identified in this review, with domains aligned to existing AI governance principles [83]. It prioritizes safety, transparency, and integration, ensuring that technological capability does not outpace ethical responsibility.”

**C—Clinical Validation.** Safety in CV prevention dictates that LLMs must never rely solely on their internal parameters for clinical advice, as this renders them prone to hallucination [36]. Systems must employ RAG to anchor responses to versioned, authoritative sources [37,57]. Furthermore, validation metrics must evolve beyond multiple-choice accuracy. Models must be tested against “Gold Standard” clinical vignettes specifically designed to stress-test performance in multimorbid and complex scenarios, where standard algorithms frequently fail to account for competing risks [54,57].

**A—Auditability.** The “black box” nature of neural networks is incompatible with clinical accountability [20]. To ensure auditability, every clinical assertion generated by an LLM must include a direct citation to the source text [37]. Additionally, institutions must maintain “Human-in-the-Loop” logs of prompts, outputs, and subsequent clinician edits. These logs serve a dual purpose: they act as an audit trail in the event of adverse outcomes and provide a feedback dataset for continuous quality improvement [39,62].

**R—Risk Stratification of Tasks.** Not all AI tasks carry equal consequences. Deployment should follow a risk-tiered “traffic light” model [74,83]:

- Low Risk (Green): Tasks such as drafting discharge summaries or patient education letters, where errors are easily detected by the patient or clinician.
- Medium Risk (Orange): Clinical decision support, such as suggesting risk factors extracted from notes, which requires mandatory clinician verification.
- High Risk (Red): Autonomous actions, such as initiating medication changes or auto-signing orders. Currently, these tasks remain prohibited for generative AI due to the risk of error.

LLMs should act as “reasoning engines” that provide options and rationales, reserving the final decision for the human clinician. Under this human-in-the-loop paradigm, ultimate professional responsibility remains with the verifying clinician, though legal frameworks for AI liability in healthcare continue to evolve.

**D—Data Privacy.** The widespread transmission of sensitive patient health information to public cloud-based models raises significant privacy and data sovereignty concerns [17,68]. To mitigate this, healthcare systems should prioritize the deployment of small language models and AI solutions that run locally within hospital firewalls [85,86]. This approach brings the model to the data, rather than sending data to the model, ensuring compliance with strict privacy regulations while reducing latency [40,85,86].

**I—Integration into Workflow.** To overcome the “implementation gap,” LLM tools must reduce, not increase, the clinician’s cognitive load [4]. Integration must move beyond separate browser tabs to be fully embedded within the EHR infrastructure [42,62]. The interaction model should shift from a “Pull” system, where the user must actively query the AI, to a “push” system, where the LLM automatically flags care gaps during the routine chart review [8,27].

**O—Ongoing Vigilance.** Clinical validation is not a one-time event [51]. Systems require continuous post-deployment monitoring to detect “model drift” or performance degradation over time [39,83]. Furthermore, as medical knowledge evolves, models suffer from temporal obsolescence. Governance protocols must ensure that the underlying knowledge bases are updated dynamically as new guidelines or pivotal trial results are published, preventing the propagation of outdated medical advice [37,55].

## 7. Narrative Review Limitations

This narrative review has inherent methodological characteristics that should be considered when interpreting its findings. Evidence selection relied on author expertise rather than systematic screening protocols, which may introduce selection bias. Formal quality appraisal was not performed given the heterogeneity of included evidence (technical benchmarks, observational studies, position papers, and conceptual frameworks), for which no single appraisal tool is appropriate. The restriction to English-language publications may limit generalizability to non-English-speaking contexts.

## 8. Conclusions

LLMs offer a realistic opportunity to bridge the gap between guideline recommendations and clinical practice in preventive cardiology. By processing unstructured clinical data, they can identify high-risk patients, synthesize complex histories and personalize health communication, extending specialist-level expertise into routine care. Current models, limited by hallucinations, outdated knowledge, and non-deterministic behavior, are unsuitable as autonomous decision-makers and are best used as “reasoning engines” to support clinicians within supervised workflows. Realizing their potential requires prospective, outcome-focused evaluation and structured governance frameworks, such as the C.A.R.D.I.O. model, to ensure safety, auditability, workflow integration, and equity. Ultimately, their value will depend on demonstrable improvements in risk factor control and CV outcomes across diverse populations.

## Figures and Tables

**Table 1:** Key technical concepts of large language models for the clinician.

Key Concept	Concise Definition	Clinical Implication
Large Language Model (LLM)	A deep learning model trained on vast text data to predict the next word and generate human-like language	Acts as a “reasoning engine” for summarisation and dialogue but lacks true understanding; outputs are probabilistic and require verification
Context Window & Tokens	The limit on the amount of text (measured in “tokens” or word parts) a model can process at one time	Long patient histories may be truncated if they exceed the window; critical data (e.g., remote events) must be explicitly present in the input to be analyzed
Hallucination	The generation of plausible but factually incorrect information, such as invented citations or non-existent drug doses	The primary safety risk in medicine; clinicians must never rely on LLMs as authoritative sources without independent verification of claims
Retrieval-Augmented Generation (RAG)	A method that connects the LLM to trusted external sources (e.g., ESC guidelines) before generating an answer	Essential for clinical accuracy; it allows the model to cite specific sources and prevents “temporal obsolescence” (outdated knowledge)
Fine-tuning	The process of adapting a general-purpose model with specific datasets (e.g., medical journals, clinical notes).	Determines if a model “speaks medicine”; generic models may struggle with complex cardiovascular terminology compared to domain-adapted versions
Stochasticity (Non-determinism)	The inherent randomness in the model; identical questions may yield slightly different answers each time	Can compromise consistency in tasks like risk triage; for formal protocols, system settings must be adjusted to minimize variability

**Table 2:** Overview of large language models applications in cardiovascular prevention across functional domains.

Functional Domain	Key Applications	Potential Benefits	Risks & Limitations	Readiness Level *
Patient-facing	Health literacy question- answering Lifestyle and behavior coaching Simplification of medical jargon	High perceived empathy and reassurance 24/7 accessibility Personalized recommendations	Hallucinations (inaccurate or fabricated advice) Literacy mismatch (reading level too high) Limited clinical nuance in complex cases	Evaluative/Early Use
Clinician-facing	Ambient documentation Guideline synthesis (via RAG) Automated risk factor extraction	Reduced burnout & clerical burden Workflow efficiency Diagnostic “rescue” of overlooked risks	Automation bias (over-trust in AI output) Temporal obsolescence (outdated knowledge) Unclear liability for errors	Early Clinical Adoption
System-facing	Automated population phenotyping Clinical registries construction Multimodal risk prediction	Scalable population surveillance Capture of uncodified factors Optimization of specialist resources	Amplification of algorithmic bias Data privacy and sovereignty concerns Model opacity (“black box”)	Research & Pilot Phase

Abbreviations: AI: artificial intelligence; RAG: retrieval-augmented generation. * Readiness levels represent the authors’ synthesis of the maturity of each application area based on the prevailing evidence: Research/Pilot Phase (primarily retrospective analyses, feasibility studies, or prototypes); Early Clinical Adoption (limited prospective evaluation and/or early deployment in constrained settings); Evaluative/Early Use (widespread informal use but limited outcome-focused evidence). These classifications are intended as pragmatic guidance rather than algorithmic thresholds.

## Data Availability

No new data were created or analyzed in this study. Data sharing is not applicable to this article.

## Abbreviations

The following abbreviations are used in this manuscript:

ACC	American College of Cardiology
AHA	American Heart Association
AI	Artificial intelligence
API	Application programming interface
CV	Cardiovascular
CVD	Cardiovascular disease
EHR	Electronic health record
ESC	European Society of Cardiology
LLM	Large language model
NLP	Natural language processing
RAG	Retrieval-augmented generation
